# Effects of substrate binding site residue substitutions of *xynA* from *Bacillus amyloliquefaciens* on substrate specificity

**DOI:** 10.1186/s12896-018-0420-7

**Published:** 2018-02-13

**Authors:** Anil S. Prajapati, Vishakha A. Pawar, Ketankumar J. Panchal, Ankit P. Sudhir, Bhaumik R. Dave, Darshan H. Patel, R. B. Subramanian

**Affiliations:** 1 P. G. Department of Biosciences, UGC-Centre of advanced studies, Satellite campus, Sardar Patel University, Sardar Patel Maidan, Bakrol-Vadtal Road, PO Box 39, Vallabh Vidyanagar, Gujarat 388 120 India; 2 P. D. Patel Institute of Applied Sciences, Charotar University of Science and Technology (CHARUSAT), Changa, Anand, Gujarat India

**Keywords:** Xylanase, Site-directed mutagenesis, Protein engineering, Substrate specificity

## Abstract

**Background:**

The aromatic residues of xylanase enzyme, W187, Y124, W144, Y128 and W63 of substrate binding pocket from *Bacillus amyloliquefaciens* were investigated for their role in substrate binding by homology modelling and sequence analysis. These residues are highly conserved and play an important role in substrate binding through steric hindrance. The substitution of these residues with alanine allows the enzyme to accommodate nonspecific substrates.

**Results:**

Wild type and mutated genes were cloned and overexpressed in BL21. Optimum pH and temperature of rBAxn exhibited pH 9.0 and 50 °C respectively and it was stable up to 215 h. Along with the physical properties of rBAxn, kinetic parameters (*K*_m_ 19.34 ± 0.72 mg/ml; *k*_cat_ 6449.12 ± 155.37 min^− 1^ and *k*_cat_*/K*_m_ 333.83 ± 6.78 ml min^− 1^ mg^− 1^) were also compared with engineered enzymes. Out of five mutations, W63A, Y128A and W144A lost almost 90% activity and Y124A and W187A retained almost 40–45% xylanase activity.

**Conclusions:**

The site-specific single mutation, led to alteration in substrate specificity from xylan to CMC while in case of double mutant the substrate specificity was altered from xylan to CMC, FP and avicel, indicating the role of aromatic residues on substrate binding, catalytic process and overall catalytic efficiency.

**Electronic supplementary material:**

The online version of this article (10.1186/s12896-018-0420-7) contains supplementary material, which is available to authorized users.

## Background

Lignocellulose is the principal and most plentiful component of the renewable biomass produced by photosynthesis and is synthesized at an estimated rate of some 200 billion tons per year [1]. These biopolymers are the major stored carbon source in the nature and are composed of cellulose (40%), hemicellulose (20–30%) and lignin (20–30%) [2]. Complete hydrolysis of these biomass requires the coordinated activities of a number of enzymes [3]. Even though a large number of xylanases are being isolated, no single enzyme has been found to be completely suitable as it is for complete hydrolysis of lignocellulose. However, these enzymes offer a good starting point for enhancing the overall economics of biofuel production. To reduce cost in biofuel production, there is a need for enzymes with improved activity and broader substrate range [4].

Research work on lignin-degrading enzymes has been focused mainly on fungi; however, of late higher growth rate, alkali tolerant and thermostable properties have directed attention towards bacterial enzymes. Modification of bacterial enzymes through protein engineering plays an important role in the production of efficient hydrolytic enzymes used in a broad range of industries. For example, cellulase enzymes access the glucose polymer cellulose while xylanases act on xylose polymer xylan and both enzymes share a common mechanism of action [5]. This is the reason why many xylanases are found to be bi-functional in nature. Though they share a similar mechanism of hydrolytic action, their preferential substrate is different. The difference lies in the architecture of the substrate binding site consist of many aromatic amino acid residues and the orientation of amino acids in the binding pocket determines the substrate preference (Gora et al., 2013). Tryptophan (W), tyrosine (Y) and phenylalanine (F) were reported relatively higher in substrate binding sites and they act as gating residues in 71 different proteins [6]. Studies have also shown how a single aromatic residue can change the orientation of substrate binding pocket [7] which clearly indicates that the positions of aromatic residues surrounding the substrate binding site dictate the substrate preference and has the key role in the binding.

The present study aims to identify the role of key aromatic amino acid residues present in the substrate binding pocket. We analyzed the structural information for XYNA which helped in the identification of the gating residues forming substrate-binding subsites of the enzyme. Five single mutants and one double mutant including wild type xylanase genes were cloned and expressed in *E. coli.* BL21. The wild type recombinant protein was purified, characterized and analyzed in comparison with engineered enzymes. Present study represents the efficient and highly valuable recombinant and engineered enzymes capable of hydrolyzing both cellulose and xylan.

## Results

### Cloning, expression, purification and characterization of rBAxn

#### Isolation of DNA and *xynA* gene

Purified genomic DNA was used as a template to amplify the *xynA* gene. A single amplified band of ~ 747 bp size was obtained which confirmed the full-length gene amplification. The nucleotide sequence of the gene was submitted to the GeneBank (BankIt accession number KR864835.1).

#### Directional cloning and expression of genes

The purified blunt-ended PCR product was ligated with vector and transformed *E. coli* DH5α. The recombinant colonies were selected and then used for plasmid isolation. Presence of desired gene and sequence obtained was subjected to similarity studies to confirm the successful cloning of *xynA* gene. BLAST alignment showed that the sequence was 99% similar to xylanase of *Bacillus amyloliquefaciens* strain DSM7. This confirmed the sequence as *xynA* from *Bacillus amyloliquefaciens*.

#### Purification of rBAxn enzymes

**Fig. 1:**
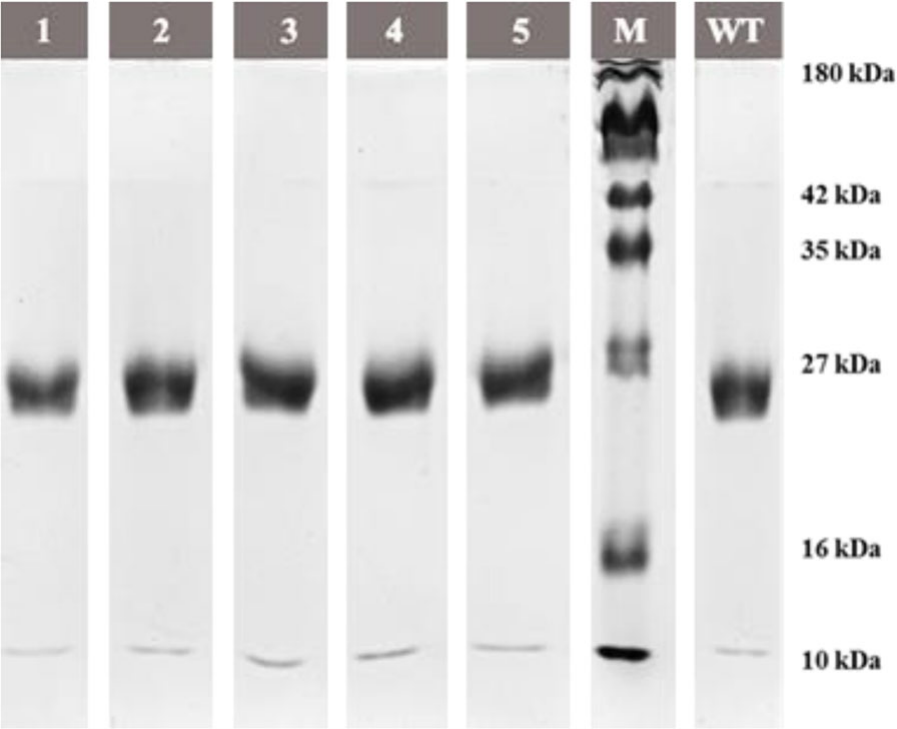
SDS-PAGE of purified recombinant engineered and non-engineered enzymes. Lane: 1 W63A mutant, Lane:2 Y128A mutant, Lane: 3 W144A mutant, Lane: 4 Y124A mutant, Lane: 5 W187A mutant, Lane: M Protein molecular weight marker (10–180 kDa), Lane: WT rBAxn

The overexpressed rBAxn was further purified to homogeneity by affinity chromatography. The pooled enzyme was concentrated and analyzed by SDS-PAGE. The molecular mass of rBAxn was confirmed alongside 4 colour pre-stained protein marker and the single band was found to be ~ 27 kDa on 12% SDS-PAGE as shown in Fig. 1. Usually, xylanases belonging to family 11 have lower molecular mass [8]. Xylanases of similar sizes were also reported from two different bacteria *Humicola insolens Y1* [9] and *Paecilomyces thermophila* cloned in *E. coli* [10]. It was observed that no band was obtained in BL21 and empty vector compared to the expressed protein.

The specific xylanase activity of lysate and pure recombinant enzyme with beechwood xylan at pH 9.0 and at temperature 50 °C exhibited 8.61 ± 0.36 IU/mg and 80.12 ± 2.52 IU/mg respectively. XYNA was purified 9.35 ± 0.69-fold by Ni-NTA chromatography with a total yield of 202.75% ± 1.40%. Commonly, family 11 xylanases are well-known to have high pI value and low molecular mass compared to family 10 xylanases which are higher molecular mass enzymes. The pI of the rBAxn recombinant enzyme was 9.25, calculated using ExPASy tool. Baek et al. (2012) [11] proposed that xylanase family with high pI and low molecular mass are considered useful for industrial applications [12]. In the present study cloned rBAxn showed a molecular mass of ~ 27 kDa only. Therefore, it could be useful in various industrial applications.

Substrate specificity of rBAxn enzyme was checked by using non-specific substrates such as CMC (Carboxymethyl cellulose sodium salt), Avicel (pH -101) and filter paper. The endoglucanase and exoglucanase activities were not detected in rBAxn recombinant enzyme.

#### Characterization of purified rBAxn enzyme

##### Effect of pH on rBAxn enzyme activity

**Fig. 2:**
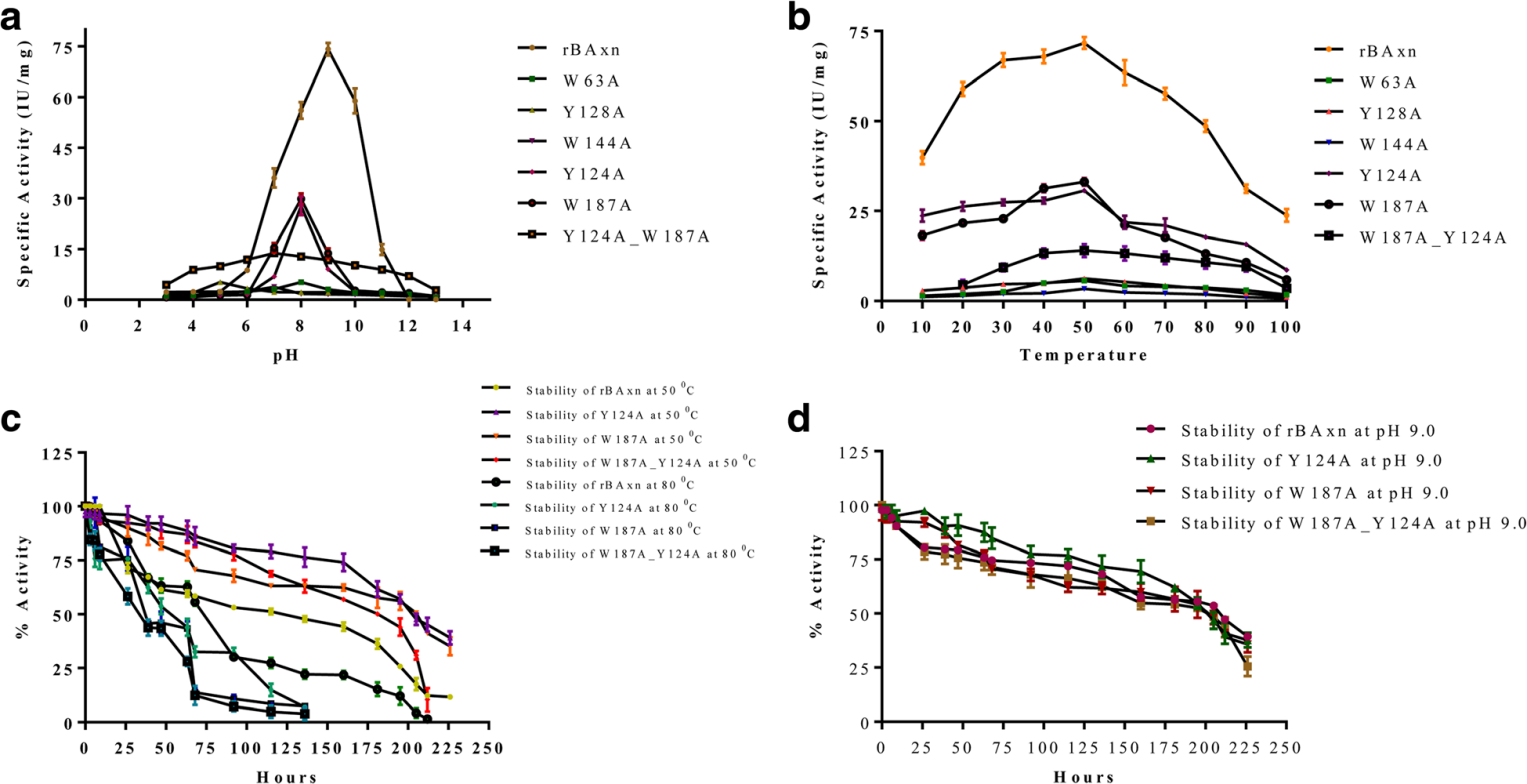
**a** Effect of pH on rBAxn and engineered enzymes activity using 1% beechwood xylan as substrate. All data are average of three individual experiments. **b** Effect of temperature on rBAxn activity using 1% beechwood xylan as substrate. All data are average of three individual experiments. **c** Stability of engineered enzymes (Y124A, W187A and W187A_Y124A) and rBAxn at 50 °C and 80 °C using 1% beechwood xylan under standard conditions. All values are expressed as mean ± SEM, based on three individual experiments. **d** Stability of engineered enzymes (Y124A, W187A and W187A_Y124A) and rBAxn at pH 9.0 using 1% beechwood xylan under standard conditions. All values are expressed as mean ± SEM, based on three individual experiments

The purified rBAxn enzyme was checked for the effect of various pH ranging from pH 3.0 to pH 13.0 as shown in Fig. 2(a). It showed good activity at pH range between 4.0 to 11.0. The maximum activity was exhibited at alkaline pH 9.0 (Fig. 2(a)) and showed relatively ~ 25% activity at pH 11.0, which shows that the rBAxn enzyme is highly active in alkaline condition. The ~ 4% activity retained at acidic pH 3.0 suggests that the rBAxn is active in acidic condition. Xylanases of many bacteria are reported to have maximum activity at or near neutral pH [13]. Alkaline enzyme is more suitable for various industrial applications.

##### Effect of temperature on rBAxn enzyme activity

The rBAxn enzyme showed maximum activity at 50 °C (Fig. 2(b)), followed by ~ 35% activity at 100 °C and ~ 57% at 10 °C which clearly indicates that the rBAxn enzyme is active in a broad range (10 °C – 100 °C) of temperatures. The enzyme also retained ~ 70% activity at 80 °C, suggesting that the rBAxn is highly thermostable and active on higher temperatures. Xylanase from *Bacillus thermoantarcticus* [14] had optimum 80 °C temperature but the optimum pH was 5.6. while in the present study the enzyme is highly active in alkaline condition, which is highly suitable for industry’s needs. Similarly, xylanase reported from *Bacillus circulans* AB 16 [15] also had an optimum temperature of 80 °C but the enzyme was not stable for more than 2 h at 65 °C.

##### Effect of various metal ions and detergents

In order to study the influence of effectors on enzyme activity, metal salts (which play an important part in the hydrolytic process) chemical reagents, modulators and detergents were used. The relative effect of effectors and detergents at 1 mM and 5 mM concentration on rBAxn enzyme activity is shown in Table 1. Among the effectors used, Cu^2+^, Ni^2+^ and Co^2+^ metal ions showed an increase in the enzyme activity at different concentrations. Baek et al. (2012) [11] also made a similar report that the presence of Cu^2+^ showed a small increase in the enzyme activity, while Mg^2+^, Fe^3+^, Pb^2+^, Hg^2+^ and Mn^2+^ metal ions and PVP detergent exhibited a decrease in the activity at higher concentration. Ca^2+^, Li^2+^, Na^+^, Zn^2+^, Cd^2+^ and K^2+^ effectors and Tween 80 and TritonX100 detergents showed no change in activity at both the concentrations which suggests that these effectors have no role in hydrolysis. Mg^2+^, Fe^3+^, and Hg^2+^ effectors, modulators, and SDS and EDTA showed the strongest inhibitory effect at 1 mM concentration. In the presence of woodward’s reagent K, NBS and PMSF at 5 mM concentration, loss of 55%, 37% and 88% showed in enzyme activity respectively which suggesting the involvement of carboxylate residues at the active site. Those effectors i.e. Ni^2+^ and Co^2+^ showed an increase in activity when used at 5 mM compared to 1 mM concentration suggesting that rBAxn enzyme is more suitable for various industrial purposes since it is active even in the high concentration of various effectors. Treatment with EDTA and SDS resulted in 50% and 80% loss in activity respectively. Inhibition by Hg^2+^ ions may be due to the interaction with sulfhydryl group [16–18] of cysteine residue (C126) and it is near to the active site. Hg^+ 2^ is also known to react with the histidine and tryptophan residues [18].

**Table 1:** Effect of Metal salts, chemical reagents and detergents on the activity of rBAxn using 1% beechwood xylan. The activity of rBAxn in the presence of 1 mM and 5 mM of each reagent was measured under standard condition. The activity without effector (no effectors in the reaction mixture) was taken as control

Metal salts, Chemicals and Detergents	Relative activity (%)^a^	
	1 mM	5 mM
Control	100	100
Mg^2+^	55.37 ± 0.51	55.35 ± 0.36
Ca^2+^	85.52 ± 0.42	106.23 ± 0.43
Ni^2+^	102.88 ± 0.91	143.77 ± 1.28
Fe^3+^	47.65 ± 0.39	45.62 ± 0.30
Pb^2+^	97.84 ± 0.34	70.28 ± 0.57
Li^2+^	96.94 ± 0.23	111.05 ± 0.84
Hg^2+^	13.77 ± 0.35	3.95 ± 0.17
Na^+^	85.40 ± 0.30	101.90 ± 1.00
Mn^2+^	80.69 ± 0.33	61.33 ± 0.56
Co^2+^	94.75 ± 0.29	123.58 ± 1.18
Zn^2+^	93.96 ± 0.24	96.34 ± 0.53
Cu^2+^	125.08 ± 0.61	116.61 ± 0.90
Cd^2+^	76.69 ± 0.89	78.16 ± 0.59
K^2+^	85.25 ± 0.35	94.80 ± 0.26
SDS	84.57 ± 0.42	12.67 ± 0.33
EDTA	40.93 ± 0.19	48.37 ± 0.47
PVP	91.77 ± 0.38	80.21 ± 0.54
Tween 80	103.10 ± 0.66	101.84 ± 0.93
Triton X 100	109.03 ± 0.68	113.39 ± 0.92
n-Bromosuccinimide	78.26 ± 0.46	63.74 ± 0.23
PMSF	23.89 ± 0.36	12.36 ± 0.89
Woodward’s reagent K	71.96 ± 0.54	45.52 ± 0.21

^a^The specific activity (in IU/mg) of control is 79.30 ± 0.82. All values are expressed as mean ± SEM, based on three individual experiments

##### Stability of rBAxn enzyme

The half-life and pH stability of rBAxn enzyme was checked and the enzyme was stable for 9 days (210–216 h) and retained 80% activity up to 50 h in pH 9.0 buffer. The enzyme showed 50% activity for 5 days (115–120 h) and for 15 h it exhibited almost 80% activity at 50 °C while 50% activity for 3 days (68–70 h) and for 26 h it exhibited almost 80% activity at 80 °C (Fig. 2(c)).

##### Determination of kinetic parameters

The kinetic properties of the rBAxn enzyme were studied at various substrate concentrations. The *K*_m_ and *V*_max_ value of wild type recombinant enzyme was 19.34 mg/ml and 318.10 μmol/min/mg respectively. The lower *K*_*m*_ value indicates high affinity of enzyme towards its substrate. Parameters like *k*_cat_ (6449.12 min^− 1^) and *k*_cat_*/K*_m_ (333.83 ml min^− 1^ mg^− 1^) were also investigated with beechwood xylan as a substrate.

Since rBAxn recombinant enzyme showed desirable properties of industrial use, we studied the sequence and structural properties of the enzyme and investigated the role of aromatic residues in the substrate binding pocket.

### Sequence analysis and homology studies

Deduced amino acid sequence (249 amino acids) of *xynA* (UniprotKb ID: E1UUS4) from *Bacillus amyloliquefaciens* was compared and aligned with the available sequences (Additional file 1: Figure S1). The sequence alignment showed conservation of amino acid residues, especially aromatic residues across the family of xylanase enzymes. Xylanase of *Bacillus subtilis* displayed almost 38.43% sequence identity and exhibited 98 identical positions. Sequence domain of both the organisms belongs to Glycosyl hydrolases family 11, involved in endohydrolysis of (1, 4)-β-D-xylosidic linkages in xylans. Based on the reported active site residues and related structures of homologous protein available in the PDB, two functional active site residues viz.: E226 and E137 were identified.

**Fig. 3:**
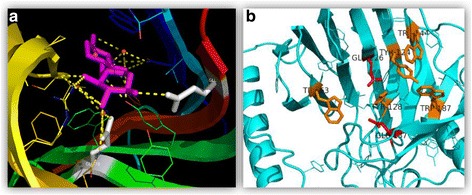
**a** Xylanase of *Bacillus subtilis* in substrate bound state with active sites and substrate binding residues, PDB (ID: 2B46). Magenta color shows the bound substrate; Gray color shows stick of active site residues. Gating residues also observed in the binding pocket. **b** 3-D model of predicted xylanase structure of *Bacillus amyloliquefaciens* obtained from I-TASSER and used for the selection of gating residues. The structure was analyzed in PyMOL, Benzene rings show the line structures of aromatic amino acids. Orange color shows targeted amino acids for the single mutations (W63, Y128, W144, Y124 and W187), Red color displays active site residues (E137 and E226)

A closer look at substrate binding pocket of XYNA of *Bacillus amyloliquefaciens* enzyme revealed the relative occurrence of aromatic amino acid present in substrate binding pocket. Sequence studies showed *Bacillus subtilis* having 36 aromatic amino acid residues while *B. amyloliquefaciens* having 33 aromatic residues. Based on the reported structure of the enzyme (xylanase of *Bacillus subtilis*) in a substrate bound state (Fig. 3(a)) in the PDB (ID: 2B46), using PyMoL [19] we identified aromatic amino acids for point mutations which could lead to a change in substrate binding properties of the enzyme.

### Model construction of mutated genes

Models of the target protein obtained using I-TASSER [20] was used to explore the amino acid sites which play an important role in binding with the substrate. In XYNA, it was also found that predominantly aromatic amino acid residues are located around the substrate binding site. By using PyMOL [19], a set of models which possess one mutated residue each were constructed. In Fig. 3(b), two catalytic residues are shown in red color and the targeted aromatic residues are shown in orange color.

### Mutagenesis

Five highly conserved non-polar aromatic amino acids (W63, Y128, W144, Y124 and W187) were targeted for substitution with alanine. Alanine is also a non-polar amino acid with a non-reactive side chain [21] which is rarely involved in protein function. As alanine is less bulkier than aromatic amino acid residues it offers less steric hindrance. In a similar work, Diao et al. (2015) used alanine residue to mutate lipoxygenase for better thermostability [22]. The fragments of *xynA* gene harboring mutation were generated by using a combination of primers i) Forward & F1R; ii) F2F & Reverse. As the amino acid was replaced, the forward primer pair carrying the specific mutation should amplify the first fragment whereas the reverse primer pair should amplify a region of second fragment, thus giving a total amplicon size of ~ 747 bp on overlap. As predicted, all mutations resulted in full length amplicons without any non-specific amplifications.

### Cloning, expression, purification and characterization of engineered enzymes

Purified mutated full-length genes were successfully cloned and confirmed through sequencing. The sequence obtained was checked for the change in nucleotide by aligning the sequence using Needle (EMBOSS) (Data not shown). DNA sequences with confirmed mutations were expressed in *E. coli.* (BL21). The production of engineered recombinant enzyme and its purification was carried out as described for the wild type rBAxn recombinant enzyme. The homogeneity of purified enzymes was checked on 12% SDS-PAGE. The molecular mass of each engineered enzymes was confirmed as shown in Fig. 1 and in each case the single band was found to be of ~ 27 kDa.

### Substrate specificity

The engineered enzymes were checked for their substrate specificity using CMC (Carboxymethyl cellulose sodium salt), Avicel (pH -101) and filter paper as substrates. In case of single mutants, the activity observed with avicel and filter paper was not remarkable while double mutant showed significant specificity with avicel and FP.

## Discussion

Thermostability is an important property for enzymes used in industries. Since rBAxn is highly stable in alkaline pH and high temperature, this enzyme could be used in bio-bleaching of pulp and other similar industries. Many of the recombinant enzymes reported, despite having a higher affinity towards their substrate were found to be not suitable for industrial applications due to a plethora of reasons. The *K*_m_ and *V*_max_ reported for xylanases by many researchers from various microorganisms are as follows: *Glaciecola mesophila* KMM241 [23] 5.82 mg/ml; 0.38 mmol/min/mg, *Thermus brockianus* [24] 3.0 mg/ml; 1324 μmol/min/mg, *Enterobacter sp.* 3.3 mg/ml; 5000 μmol/min/mg, *Malbranchea pulchella* [25] 4.6 mg/ml; 82 μmol/min/mg, *Bacillus licheniformis* 6.7 mg/ml; 379 μmol/min/mg, *Bacillus pumilus* [26] 4.0 mg/ml; 5000 μmol/min/mg for Xyl 1 and 3.5 mg/ml; 3448 μol/min/mg for Xyl 2. Very few reports are available on *Bacillus sp.* with low *K*_m_*,* by Baek et al. (2012) [11] 0.363 mg/ml of XYNA His, *B. sterothermophilus* [27] 1.63 mg/ml, *Bacillus sp. SPS-0* [28] 0.7 mg/ml. Though the affinity of these enzymes is high, other properties of the enzymes such as thermostability, alkalinity and specificity diminished their suitability for commercial use.

The activity of purified engineered enzymes was checked and compared with rBAxn (non-engineered recombinant enzyme) (Summarized in Table 2) using 1% beechwood xylan. All five engineered recombinant enzymes showed reduction in specific activity in comparison to rBAxn. Nearly 8% activity was retained in W63A and Y128A while only 5% activity was retained in W144A. However, Y124A and W187A showed nearly 40–45% specific activity. In a similar report exchange of Trp to Ala at 31 and 218 in human chitotriosidase showed 12-fold reduction in degradation [29]. Decreased specific activity with specific substrate (xylan) of rBAxn revealed the importance of aromatic residues in substrate binding as well as determining the catalytic efficiency of the enzyme.

**Table 2:** Specific activities of engineered and non-engineered enzymes using 1% beechwood xylan, 2% CMC, 2% Avicel and Whatman filter paper as substrate. – activity not detected

Sr. No.	Enzyme	Relative Specific Activity (%)^a^	Specific activity (IU/mg)		
		Xylan	CMC	Avicel	FP
1.	rBAxn	100	–	–	–
2.	W63A	7.5	0.139 ± 0.0017	–	–
3.	Y128A	8.02	0.214 ± 0.0023	–	–
4.	W144A	5.4	0.210 ± 0.0015	–	–
5.	Y124A	40.7	0.140 ± 0.0012	–	–
6.	W187A	44.2	0.247 ± 0.002	–	–
7.	W187A_Y124A	19.7	0.289 ± 0.025	11.23 ± 0.029	1.97 ± 0.023

^a^The specific activities (in IU/mg) of rBAxn on xylan is 80.12 ± 2.52. All values are expressed as mean ± SEM, based on three individual experiments

The mutant enzymes also showed a shift in their optimum pH from pH 9.0 as shown in Table 3, while the optimum temperature of mutant enzymes did not show any change indicates that these single mutations have no role in reaction temperature. Comparatively, low specific activity was observed using beechwood xylan (specific substrate) in all five mutants. Out of five mutants, two Y124A and W187A were selected to check their kinetic parameters and stability at optimum pH 9.0 and 50 °C. Interestingly both the mutants sustained their stability which shows that the selected aromatic residues have no role in the stability of the enzymes at maximum temperature and pH. Kinetic properties of Y124A and W187A, are shown in Table 4. The *K*_m_ of both the enzymes are changed. Interestingly, results indicate that the substituted residues (aromatic amino acids) are important for binding with xylan. The results clearly show that the substituted aromatic amino acids are important for substrate binding as is evident from the increase in *K*_m_ value of both mutant protein. Replacement of aromatic amino acids such as W85A, Y172A and W274A in xylanase A of *Streptomyces lividans* also showed an increase in *K*_m_ value as compared to wild type [30]. This clearly indicates the role of aromatic residues towards substrate binding. Kristine et al., also reported on the role of aromatic residues in substrate degradation [29] which were important for catalytic activity. Out of five single mutations, two were selected to carry out for the double mutation based on the activity and alteration in the substrate binding. Y124A_W187A double mutant was also cloned, expressed and its properties were compared with rBAxn and single mutants. The properties of engineered and non-engineered enzymes are summarized in Tables 3 and 4 with different substrates.

**Table 3:** Physical properties of rBAxn and engineered enzymes using 1% beechwood xylan and 2% CMC as a substrate. – Activity not detected

Enzymes	Xylan		CMC	
	pH	Temperature	pH	Temperature
rBAxn	9	50	–	–
W63A	8	50	5	50
Y128A	5	50	5	50
W144A	7	50	5	50
Y124A	8	50	5	50
W187A	8	50	8	50
W187A_Y124A	7	50	5	50

**Table 4:** Kinetic parameters of rBAxn and engineered enzymes using 1% beechwood xylan as a substrate

Sr. No.	Enzymes	*K*_m_ (mg/ml)	*K*_cat_ (min^−1^)	*K*_cat_/*K*_m_ (ml min^−1^ mg^−1^)
1.	rBAxn	19.34 ± 0.72	6449.12 ± 155.37	333.83 ± 6.78
2.	W63A	*	*	*
3.	Y128A	*	*	*
4.	W144A	*	*	*
5.	Y124A	23.92 ± 0.10	823.58 ± 3.12	34.81 ± 0.19
6.	W187A	140.36 ± 1.15	4258.08 ± 89.73	28.75 ± 0.61
7.	W187A_Y124A	45.75 ± 0.23	1080.65 ± 23.65	23.62 ± 0.26

*not performed. All values are expressed as mean ± SEM, based on three individual experiments

All the engineered enzymes showed good specific activity as shown in Table 2. Similarly, Ichinose in 2012 designed mutants of SoXyn10A from *Streptomyces olivaceoviridis* E-86 to discriminate the xylose and glucose based substrate [31] and tried to remove steric hindrance. They reported that Gln88 and Trp274 were needed to be moved to accommodate the extra C6 hydroxyl [31]. The physical properties of enzymes were also checked using CMC as a substrate, and it showed acidic pH for optimum activity at a temperature similar to that of rBaxn. One of the interesting observations in the present study is that replacement of aromatic amino acids by a simple side chain amino acid, shifts the pH from alkaline to acidic. In case of double mutant, enzyme activity with avicel, CMC and FP indicate that replacement of aromatic residues in the substrate binding pocket has resulted in flexibility towards other substrates.

Two mutations, W187A and Y124A retained 40–45% activity compared wildtype while W63A, Y128A and W144A which lost 90% activity. Compared to other mutants, Y124A differs mainly by large decrease in *k*_cat_ suggesting that it is involved not only in substrate binding but also in the catalysis step. Generally, aromatic amino acids are important for sugar binding proteins and also for the thermal stability of the enzymes [30]. Replacement of these residues showed significant results and make specific enzyme multi-functional. Enzyme cocktails for bioethanol production are cost drivers [31]. Ichinose in 2012 also suggested that a multifunctional enzyme would be desirable for the cheap and effective hydrolysis of biomass.

## Conclusions

One of the most significant property of industrially important enzymes is their thermostability. In this study, the rBAxn enzyme was highly thermostable and showed optimum activity with in alkaline conditions, active in the presence of many salts, detergents and chemicals. Engineered enzymes apart from showing activity on specific substrate, also showed activity on non-specific, more crystalline and larger substrate. This shows that, the steric hindrance at binding pocket of enzyme has been reduced by substitution of aromatic amino acids with alanine which further suggests that the selected residues act as gate keepers for other substrates. For biofuel production, lignocellulose is the best substrate, but no single enzyme can perform complete degradation of crystalline lignocellulose. This problem could be solved by using engineered enzymes like Y124A, W187A and W187A_Y124A which showed bifunctional activity on different substrates compared to rBAxn enzyme.

## Methods

### Bacterial strains and vector

Bacterial strain, *Bacillus amyloliquefaciens* 1270 was procured from MTCC (Microbial Type Culture Collection), Chandigarh, India. The culture was revived on Nutrient media at 30 °C. For cloning and expression, the *E. coli* (DH5α) and *E. coli* (BL21) were used respectively. The *E. coli* strains were grown on LB (Luria-Bertani) medium supplemented with 100 μg/ml of ampicillin at 37 °C. The pET101/D-TOPO (5753 bp) Vector was used (Invitrogen, USA). Blunt end PCR products were used for directional cloning using Champion™ pET Directional TOPO^®^ Expression Kit (Invitrogen, USA).

### Isolation of DNA and cloning of x*ynA* gene

DNA was isolated from *Bacillus amyloliquefaciens* 1270 using standard method [32]. Primers were designed for *xynA* gene manually, sequence used for primer designing was taken from NCBI nucleotide database. Gene coding xylanase was amplified with help of synthesized primer pairs (IDT, USA) *xynA*_FP (CACCATGACGGTAAGACCTCAATAC) and *xynA*_RP (AAAGGATTTTCCGCTAATAGTCAG). The polymerase chain reaction contained 0.4 μM forward and reverse primers, 2.0 μl of template DNA, 5 μl of 10X high fidelity PCR buffer, 2.0 mM MgSO_4,_ 0.2 mM deoxy nucleotide solution mix, 1 U high fidelity platinum Taq DNA polymerase. Total volume of reaction mix was 50 μl made with Milli-Q. Amplification condition started with initial denaturation at 94 °C for 2 min, denaturation for 15 s, followed by annealing at 56.2 °C for 30 s, extension for 1 min at 68 °C and final extension was for 10 min at 68 °C. The amplified product was purified and used as insert for cloning in a pET101/D-TOPO vector. The ligated product was transformed using standard method for transformation.

### Gene expression and purification

Recombinant clone (rBAxn) was expressed in *E. coli* (BL21) cells by growing them under shaking condition at 37 °C in 100 μg/ml ampicillin containing AIM-Super growth medium (alternate for IPTG-inducible expression) with trace elements (Hi-Media). Overnight grown cells were harvested by centrifugation at 7800 g for 10 min [33]. Cell pellet was suspended in lysis buffer (pH 8.0) containing 300 mM NaCl and 50 mM Tris-Cl. The Suspended pellet of cells were lysed by using an ultrasonicator (Vibra cell, Sonics, USA) to release the intracellular recombinant xylanase. Cell suspension was disrupted by sonicator probe with pulses at a 20 s on and 04 s off cycles for 5 min, amplitude was set up to 30%. Heat generation during sonication was maintained by providing cooling condition with crushed ice. The cell lysate thus obtained was centrifuged at 7800 g for 15 min at 4 °C. The cell free supernatant was used as a crude enzyme. The recombinant enzyme was purified in a single step using Ni-NTA (Nucleo-pore, Genetix, India) affinity chromatography. His-tagged recombinant xylanase was purified by different steps of purification such as equilibration, washing and elution steps. Initially, column was equilibrated with pH 8.0 lysis buffer (50 mM Tris-Cl and 300 mM NaCl). After equilibration, crude sample was added followed by wash buffer (50 mM Tris-Cl, 300 mM NaCl and 20 mM Imidazole). Fractions were collected by addition of elution buffer (50 mM Tris-Cl, 300 mM NaCl and 250 mM Imidazole). The recombinant protein (His-tagged) was eluted with an imidazole gradient. Eluted fractions having proteins were monitored by the determination of protein concentration by Bradford’s method [34]. Collected enzyme fractions were concentrated and extra salts were removed by using 10 kDa filter (Amicon, Millipore).

### Molecular mass determination

Molecular mass of purified proteins was determined by polyacrylamide gel electrophoresis (SDS-PAGE). The separation of protein was carried out in 1.0 mm slab gel of 12% polyacrylamide (*w*/*v*) [35]. Protein sample was denatured at 95 °C for 10 min. After electrophoresis, the protein bands were stained with Brilliant Blue R (Sigma-Aldrich). Approximate molecular mass of recombinant enzyme was determined using 4 color pre-stained protein ladder (Puregene, Genetix).

### Enzyme activity assay

In order to determine the activity of rBAxn and engineered enzymes, the reaction mixture contained 50 μl of 1% *w/v* beech wood xylan (Sigma, Aldrich) prepared in 0.2 M glycine-NaOH buffer (pH 9.0), 50 μl diluted enzyme and 100 μl buffer (Gly-NaOH, pH 9.0). Reaction system was incubated at 50 °C for 15 min. The released sugars were estimated using DNS (3,5-dinitrosalicylic acid) method [36] using xylose as a standard followed by final 1.2 ml volume made up with distilled water which was used to take absorbance at 540 nm. One unit (U) of xylanase activity was defined as the amount of enzyme required to release 1 μmol of xylose (reducing sugar) in 1 min under the mentioned conditions.

### Enzyme characterization

Properties of recombinant enzyme such as pH, temperature, stability at maximum pH and temperature, thermostability, kinetic parameters, effect of metal ions and detergents on enzyme activity and bi-functionality were checked.

#### Effect of pH on rBAxn activity

Different pH buffers were prepared according to standard methods of biochemical analysis [37]. The pH ranges from 3.0–5.0, 6.0–8.0 and 9.0–13.0 were made using Citrate Buffer (0.1 M), potassium phosphate buffer (0.1 M) and Glycine-NaOH buffer (0.1 M) respectively. The effect of pH on rBAxn was determined using pH range 3.0 to 13.0 at 50 °C.

#### Effect of temperature on rBAxn activity

The effect of temperature on rBAxn was determined by incubating the reaction mixture with enzyme at temperature from 10 °C to 100 °C. The reaction mixture contained 0.1 M Glycine-NaOH buffer of pH 9.0 and 1% xylan as a substrate.

#### Effect of metal ions and detergents

Various effectors at concentrations of 1 mM and 5 mM were used to evaluate their effect on enzyme activity. Chemical reagents, modulators (n-Bromosuccinimide, PMSF (Phenylmethylsulfonyl fluoride), Woodward’s reagent K) and metal ions such as Ca^2+^, Mg^2+^, Fe^2+^, Cu^2+^, Mn^2+^, Co^2+^, Zn^2+^, Hg^2+^, K^+^, cd^2+^, Ni^2+^, Pb^2+^, Na^+^ and Li^2+^ were prepared. To determine the effect of detergent on enzyme activity, different detergents such as SDS (Sodium dodecyl sulfate), PVP (Poly Vinyl Pyrrolidone), Triton 100 and Tween 20 were also prepared. Solution of salts/detergents were added to the enzyme to obtain a final concentration of 1 mM/5 mM. This mixture was incubated at room temperature for 60 min. The activity of rBAxn was measured at optimum condition at pH 9.0 and 50 °C.

#### pH and temperature stability

The stability (at maximum pH and temperature) of rBAxn was evaluated by incubating the enzyme at 50 °C with and without 0.1 M Gly-NaOH buffer, pH 9.0. For temperature stability, the enzyme was incubated without any buffer while in case of pH stability, the enzyme was incubated with buffer at pH 9.0. 1% *w/v* xylan was added after different time intervals and the reaction mixture was further incubated for 15 min to allow the catalytic reaction under standard conditions. The reducing sugar was estimated by stopping the reaction by addition DNSA reagent.

#### Determination of kinetic parameters

Substrate saturation curve was plotted by calculating the activity of rBAxn with a range of substrate concentrations from 10 to 20 mg in 0.1 M Gly-NaOH (pH 9.0) buffer at 50 °C. The kinetic parameters *K*_*m*_ and *V*_*max*_ were determined by Line weaver-Burk plot according to MM equation.

Bi-functionality of rBAxn was also checked replacing xylan with CMC (Carboxymethyl cellulose sodium salt, medium viscosity 200–300 CPS, chemoport, India), Whatman filter paper and Avicel (pH -101, Sigma-Aldrich) as substrates.

### Mutagenesis

The *xynA* gene (747 bp) of *Bacillus amyloliquefaciens* 1270 was used as a template in the study. Nucleotide sequence of *xynA* was analyzed using various computational tools and online available softwares such as EMBOSS [38], BLAST (ExPASy), ClustalW2 (EBI). For protein sequence analysis, Compute pI/Mw (ExPASy) and AACompIdent (ExPASy) tools [39] were used. Based on the Homology modelling of available protein structure in Protein Data Bank (RCSB PDB) and sequence similarity, sets of various probable gating residues were identified for single mutations.

The site specific single mutations were targeted for *xynA* gene. Pairs of fragments were generated using primer pairs (Additional file 1: Table S1) which were designed manually (IDT, USA). The full length mutant genes were obtained using mutated fragments by overlap extension PCR method [40, 41]. The full length mutated genes were amplified (same condition mentioned above) and its library was generated.

### Cloning, expression, purification and characterization of mutants

Mutated amplicons were used as inserts for cloning in a pET101/D-TOPO vector. The ligated products were transformed using standard method for transformation. All mutated recombinant clones (W63A, Y128A, W144A, Y124A and W187A) were expressed in *E. coli* (BL21) cells by growing them at 37 °C in 100 μg/ml ampicillin containing AIM-Super growth medium with trace elements at 120 rpm. The overexpression and purification methods were same used for wild type recombinant enzyme (rBAxn).

### Biochemical characterization of engineered enzymes

All purified mutant enzymes were characterized by estimating their optimum pH, temperature, stability of enzymes and kinetic parameters compared to the wild type recombinant (rBAxn) enzyme. The standard assay conditions and methods were same as mentioned in the characterization of rBAxn section.

### Substrate specificity

The purified engineered proteins were assayed against various substrates known for cellulose such as CMC, Avicel, filter paper. The assay method was same as used for the xylanase assay (0.1 M Gly-NaOH buffer, pH 9.0 at 50 °C) but the substrates were replaced with xylan and the incubation time was 60 min. All engineered proteins were compared with rBAxn.

## Additional file

Additional file 1: **Table S1.** Primer sequences used for the fragment generation. **Figure S1.** BLAST analysis and sequence alignment of xylanase with available online database using UniprotKb alignment tool. Red color shows reported active site residues and yellow color displays the location of active residues present in the XYNA of *Bacillus amyloliquefaciens*. Orange color indicates conserved aromatic residues across the family of xylanase enzymes. The black color boxes display the selected aromatic amino acid residues for single mutation in XYNA of *Bacillus amyloliquefaciens*. (DOCX 1540 kb)

## Abbreviations

GH11: Glycosyl hydrolase family 11
kcat: turnover number
Km: Michaelis constant
LB: Luria-Bertani
MTCC: Microbial Type Culture Collection
PDB: Protein Data Bank
PMSF: Phenylmethylsulfonyl fluoride
rBAxn: Wild type recombinant xylanase enzyme
Vmax: Maximum velocity
*xynA*: Xylanase gene
