# Supplementary material for: Effects of substrate binding site residue substitutions of xynA from Bacillus amyloliquefaciens on substrate specificity
Source: BMC Biotechnol. 2018 Feb 13;18:9. doi: 10.1186/s12896-018-0420-7 (PMC5812043; doi:10.1186/s12896-018-0420-7)
Supplement: Supplementary file 1 — Table S1. Primer sequences used for the fragment generation. Figure S1. BLAST analysis and sequence alignment of xylanase with available online database using UniprotKb alignment tool. Red color shows reported active site residues and yellow color displays the location of active residues present in the XYNA of Bacillus amyloliquefaciens. Orange color indicates conserved aromatic residues across the family of xylanase enzymes. The black color boxes display the selected aromatic amino acid residues for single mutation in XYNA of Bacillus amyloliquefaciens. (DOCX 1540 kb) [file 12896_2018_420_MOESM1_ESM.docx]

| Sr. No. | Name of Primer | Sequence |
| --- | --- | --- |
| 1 | W63AF2F | TAT GAA TTG **GCC** AAG GAC TCT |
| 2 | W63AF1R | AGA GTC CTT **GGC** CAA TTC ATA |
| 3 | Y128AF2F | CTG TGT GTT **GCC** GGA TGG ACG |
| 4 | Y128AF1R | CGT CCA TCC **GGC** AAC ACA CAG |
| 5 | W144AF2F | GTC GAT AAT **GCC** GGC ACC TAC |
| 6 | W144AF1R | GTA GGT GCC **GGC** ATT ATC GAC |
| 7 | Y124AF2F | GGG AAT TCC **GCC** CTG TGT GTT |
| 8 | Y124AF1R | AAC ACA CAG **GGC** GGA ATT CCC |
| 9 | W187AF2F | AAA CAG TAT **GCC** AGT GTC CGG |
| 10 | W187AF1R | CCG GAC ACT **GGC** ATA CTG TTT |

Table S1 Primer sequences used for the fragment generation


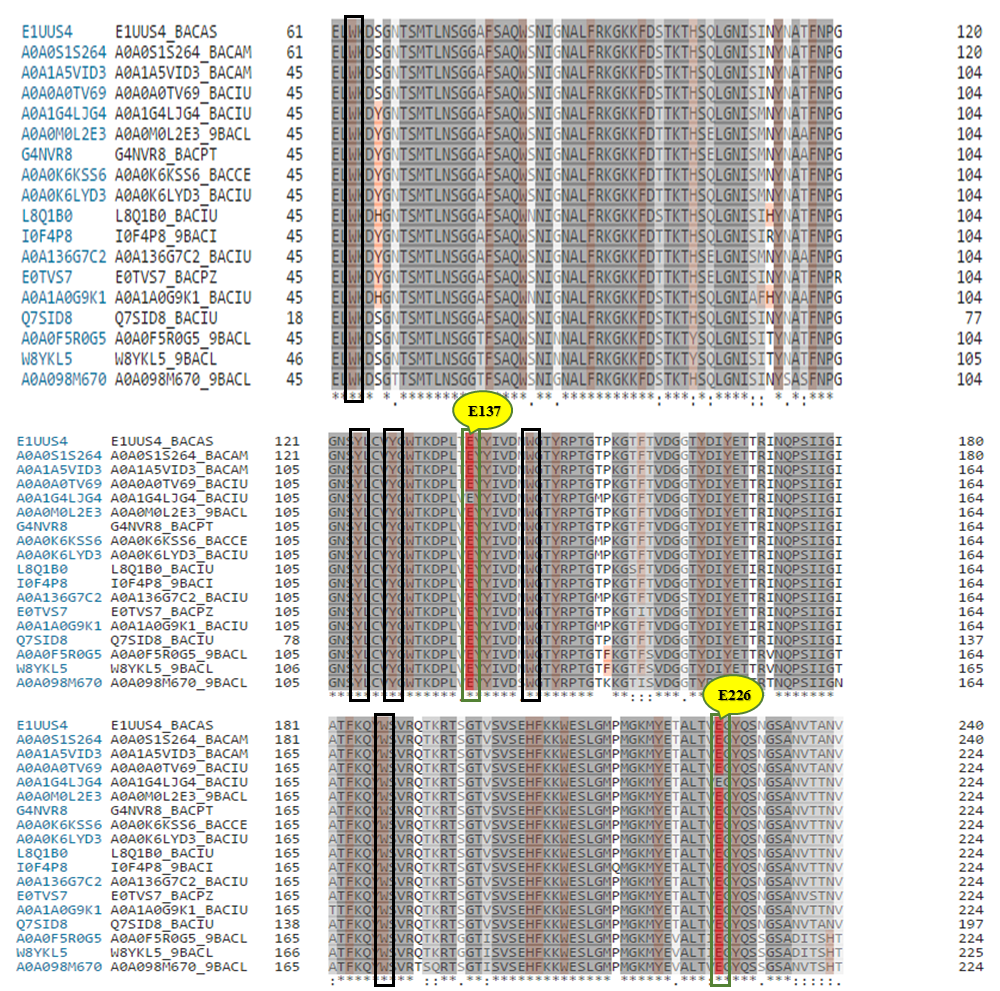
Figure S1 BLAST analysis and sequence alignment of xylanase with available online database using UniprotKb alignment tool. Red color shows reported active site residues and yellow color displays the location of active residues present in the XYNA of *Bacillus amyloliquefaciens*. Orange color indicates conserved aromatic residues across the family of xylanase enzymes. The black color boxes display the selected aromatic amino acid residues for single mutation in XYNA of *Bacillus amyloliquefaciens*.
